# Thermoregulatory response of *Frankliniella occidentalis* (Pergande) (Thysanoptera: Thripidae) to infection by *Beauveria bassiana*, and its effect on survivorship and reproductive success

**DOI:** 10.1038/s41598-019-49950-z

**Published:** 2019-09-20

**Authors:** Xiaochen Liu, Stuart R. Reitz, Zhongren Lei, Haihong Wang

**Affiliations:** 1 State Key Laboratory for Biology of Plant Diseases and Insect Pests, Institute of Plant Protection, Chinese Academy of Agricultural Sciences, Beijing, 100193 P.R. China; 2 Malheur Experiment Station, Oregon State University, 595 Onion Avenue, Ontario, Oregon 97914 USA

**Keywords:** Behavioural ecology, Population dynamics

## Abstract

Behavioral thermoregulation is a defensive strategy employed by some insects to counter infections by parasites and pathogens. Most reported examples of this type of thermoregulatory response involve behavioral fevering. However depending upon the life history of a host-insect and that of the parasite or pathogen, the host may respond by cold-seeking behavior. Thermoregulation is not only ecologically important; it may affect the success of parasites and pathogens as biological control agents. We examined if *Frankliniella occidentalis* (Pergande) thermoregulates in response to infection by *Beauveria bassiana*, a fungal pathogen commonly used for biological control. Fungal-infected thrips preferentially moved to cooler areas (~12 °C) while healthy thrips sought out warmer temperatures (~24 °C). This cold-seeking behavior suppressed the growth of *B. bassiana* in infected thrips, and significantly improved survivorship of infected thrips. At 24 °C, males only survived up to 10 d after infection and females up to 20 d after infection, which was substantially poorer survivorship than that of corresponding healthy individuals. However, individuals of both sexes survived up to 48 d after infection at 12 °C, which was a much less severe reduction in survivorship compared with the effect of *B. bassiana* infection at 24 °C. The proportion of females among progeny from infected thrips at 12 °C was higher than at 24 °C. Therefore, cold-seeking behavior is beneficial to *F*. occidentalis when infected by *B. bassiana*, and its effects should be considered in the use of *B. bassiana* in biological control programs.

## Introduction

Some insects thermoregulate in response to pathogen or parasite infection, either as a result of manipulation by pathogen or macroparasite or as a host defensive strategy^1^. Behavioral fevering is a defensive strategy used by some insects, in which an insect seeks to increase its body temperature to levels that are detrimental to parasites or pathogens.

Behavioral fevering occurs in many, diverse insect-pathogen systems because even slight temperature increases can reduce the virulence of fungal pathogens and/or the susceptibility of their insect hosts to infection. Insects known to display febrile responses to fungal infection include *Oedaleus senegalensis*^2,3^*, Schistocerca gregaria*^4,5^ and *Locusta migratoria migratorioides*^6,7^ when infected by *Metarhizium* spp., *Musca domestica* when infected by *Entomophthora muscae*^8^ or by *Beauveria bassiana*^9^, and *Apis mellifera* when infected by *Ascophaera apis*^10^. Behavioral fevering has also been observed among insects infected with microsporidia, such as *A. mellifera* when infected with *Nosema ceranae*^11^, and *Melanoplus sanguinipes* when infected with *Tubulinosema acridophagus*^12^. Behavioral fevering in response to bacterial infection has been observed in *Acheta domesticus* infected by *Rickettsiella grylli*^13^, and in *Gromphadorhina portentosa*^14^. The tenebrionid beetles *Onymacris plana* and *Tenebrio molitor* also display behavioral fevering when inoculated with bacterial lipopolysaccharides^15^. Behavioral fevering is not limited to insects infected with microparasites. Solar basking by the caterpillar *Platyprepia virginalis* reduces mortality from parasitization by the tachinid fly *Thelaira americana*^16^.

Behavioral thermoregulation can also take the form of chilling whereby infected insects actively lower their body temperature to reduce their susceptibility to infection or to suppress parasite or pathogen growth and virulence^17^. However, compared with fevering, relatively few cases of behavioral chilling have been reported. The fruit fly, *Drosophila melanogaster*, exhibits behavioral chilling in response to infection by certain fungal^18^ or bacterial pathogens^19^. Workers of the bumblebee, *Bombus terrestris*, infected with conopid fly parasitoids remain outside their nest at night, where colder temperatures retard development of the parasitoid^20^. Similarly, acanthocephalan-infected cockroaches preferentially move to cooler locations to suppress parasite development^21^.

Previous studies of thermoregulatory responses to infection have largely focused on their effects on survival, fecundity, flight capacity, or mating competitiveness of infected insects^2,3,7,18,22^. However, less attention has been paid to the effects of thermoregulatory behaviors on the ultimate reproductive success of infected individuals, including the survivorship and sex ratio of their progeny, which are important factors influencing overall reproductive success.

Western flower thrips, *Frankliniella occidentalis* (Pergande) (Thysanoptera: Thripidae), is a worldwide invasive agricultural pest that is now distributed from northern temperate zones to southern temperate zones^23^. *F. occidentalis* has an arrhenotokous reproductive mode, with males arising from unfertilized eggs and females from fertilized eggs^24^. Many haplodiploid species have sex ratios that are influenced by local conditions^25^. Kumm and Moritz (2010) demonstrated that the sex ratio of *F. occidentalis* is affected by variations in temperature, with increasing proportions of females occurring as temperatures increase^26^. Offspring of *F. occidentalis* treated with the entomopathogenic fungus *Beauveria bassiana* have a more male-biased sex ratio (0.4 ♀:1 ♂) than the progeny from untreated parents, which have an even sex ratio (1 ♀:1 ♂)^27^.

Because of its pathogenicity to *F. occidentalis, B. bassiana* has been widely used as a mycoinsecticide for the biological control of *F. occidentalis*^27^. However, *B. bassiana* infection is known to induce febrile responses in certain host insects, which can reduce the pathogenicity of the fungus^9^. Consequently, defensive thermoregulation may decrease the efficacy of *B. bassiana* as a biological control agent^28^. Therefore, we investigated whether *F. occidentalis* uses thermoregulatory behaviors in response to *B. bassiana* infection.

*Frankliniella occidentalis* is known to display thermoregulatory behaviors, in part to reduce risks from heat-induced dessication. They preferentially inhabit cooler, shady areas, such as within flowers or on the underside of leaves. Flight activity is largely restricted to cooler, early morning hours. Flight activity is also greater on rainy or cloudy days than on sunny days^29^. Given that growth of *B. bassiana* occurs under warm conditions (~30 °C) and that *F. occidentalis* needs to minimize exposure to high temperatures, we hypothesized that *B. bassiana* growth would be inhibited when host thrips are exposed to lower temperatures and that infected *F. occidentalis* would live longer when kept at cooler temperatures. In addition, we determined if thermoregulatory responses to fungal infection affect the sex ratio of thrips progeny.

## Materials and Methods

### Insect

To establish a colony, approximately 500 adult *F. occidentalis* were collected from pepper (*Capsicum annuum* L.) in Lang Fang, China, in 2015., and maintained as described by Zhang *et al*.^27^. Briefly, thrips were reared on bean pods (*Phaseolus vulgaris* L.) in an environmental chamber (MLR-351H, SANYO Electric Co., Ltd.) at 26 °C, a photoperiod of 14:10 (L:D) h, and 60–70% RH.

### Fungi

*B. bassiana* strain GZGY-1-3 (deposited in China General Microbiological Culture Collection Center no. 9254; GenBank no. KP994951 was used in all experiments. The fungal strain was maintained on Sabouraud dextrose agar at 26 °C under continuous darkness. Conidial suspensions were prepared with 0.05% Tween-80 in sterile water, according to the methodology described by Goettel & Inglis (1997)^30^ for use in bioassays. Previous tests confirm that this strain is highly virulent to *F. occidentalis* at 26 °C and a concentration of 1 × 10^7^ conidia per milliliter^27^; however, its virulence decreases with decreasing temperature^31^.

### Temperature preference of *F. occidentalis*

The thermoregulatory response of thrips was observed on a purpose-built apparatus, which was modified from Sayeed & Benzer (1996)^32^. The main body of the apparatus was an aluminum board (90 cm length × 30 cm width). One end was connected to a heating bar equipped with a thermostat (BH-3) to ensure temperature stability. The opposite end was cooled through a pumped ice-water mixture to create a uniform temperature gradient along the length of the apparatus. The aluminum board was marked into 27 equal sections, which were used to identify the position of thrips across the temperature gradient. A thermocouple (TES-1310) was used to measure the mid-point of each section across the apparatus, which confirmed a linear temperature gradient ranging from 8 to 35 °C (±0.15 °C) for a change of 0.3 °C/cm along the length of the board. Three isolated escape-proof experimental lanes were created along the length of the apparatus with perspex bars. The apparatus was cleaned with 70% ethanol before and after experimental replicate.

Adult thrips (CO_2_-anaesthetized for 3 sec) were dipped into a conidial suspension (infected) or sterile water containing 0.05% Tween-80 (healthy control) for 5 sec. After treatment, thrips were allowed to dry on filter paper and then were transferred to Petri dishes (diameter: 7 cm). Petri dishes were provisioned with a fresh bean pod, and covered with parafilm that had been pricked with a needle for ventilation. Based on preliminary studies to optimize fungal infection, Petri dishes were stored in chamber at 24 °C, RH 60–70%, and 14 L: 10 D photoperiod.

Twenty-four hours after treatment, thrips were collected in glass bottles (6 cm high by 2 cm in diameter). Thrips were then released at the temperature point of 24 °C in each lane of the gradient. Based on preliminary experiments, the position of thrips along the temperature gradient was recorded by photography after 16 minutes of exposure. Thrips that stood on an interval line were counted as being in the lower temperature zone. Temperature preferences were always assessed between 14:00 and 16:00^29^, and the experiment was carried out in a room with a uniform light source, assuring there was no confounding effect of light.

### Effects of chilling behavior on the survival, fecundity and sex ratio of thrips

To infect thrips, pairs of thrips (1 day old adults, 1 ♂: 1 ♀) were dipped for 5 seconds at a spore suspension 1 × 10^7^ conidia per milliliter. Healthy, control thrips were handled in a similar manner, except that they were dipped in sterile water containing Tween-80 at 0.05%. Thrips were allowed to dry on filter paper and then subsequently transferred to glass cylinders (10 cm in diameter and 27 cm in height). The ends of each cylinder were covered with fine mesh cloth (200 mesh) for ventilation, and the cylinders were provisioned with one bean pod each as a food source and oviposition substrate. Containers were stored in an environmental chamber at 12 °C (preferred temperature of *B. bassiana*-infected thrips) and 24 °C (preferred temperature of healthy thrips), RH 60–70%, and 14:10 L:D photoperiod. Thrips survival was scored daily until all thrips died (n = 12 cylinders, 20 pairs of thrips/cylinder, 3 cylinder replicates/treatment).

Dead thrips were removed and placed on filter paper moistened with sterile water in sealed Petri dishes (approximately 7 cm in diameter), at 26 ± 1 °C for up to 5d. These thrips were examined daily for signs of *B. bassiana*-like fungal growth to determine if death resulted from mycosis^27^. The sex of surviving thrips was recorded daily.

To determine oviposition rates, bean pods were replaced daily. Each day, bean pods were removed from the adult thrips and isolated in new glass cylinders (3 cm in diameter and 19 cm in height). Ends of the containers were covered with fine mesh cloth (200) for ventilation, and all of these containers stored in an environmental chamber at 24 °C. The numbers of first instars emerging from each pod were counted as a measure of daily fecundity^33^. To determine the sex ratio of offspring, we transferred each newly eclosed first instar to a Petri dish (3.5 cm in diameter) containing a 2-cm-length bean pod and covered with parafilm, which was pricked for ventilation. Bean pods were replaced every day until thrips died or eclosed as adults.

### *Beauveria bassiana* content in infected thrips

To examine the effect of temperature on the growth of *B. bassiana* in thrips, we analyzed the *B. bassiana* content of infected thrips over time when reared at 12 °C, the temperature preferred by infected thrips, and at 24 °C, the temperature preferred by healthy thrips.

#### Sampling of infected thrips for *B. bassiana* levels

To determine levels of *B. bassiana* over time in infected thrips, groups of mixed sex thrips (n = 2000) were dipped for 5 seconds in a conidial suspension (1 × 10^7^ conidia per milliliter). After treatment, thrips were allowed to dry on filter paper and then were transferred to Petri dishes (diameter: 7 cm) provisioned with a fresh bean pod and covered with parafilm, which was pricked with needle for ventilation. Petri dishes were stored in environmental chambers at 12 °C or 24 °C, RH 60–70%, and 14:10 L:D photoperiod, respectively.

The thrips treated with fungi were collected for DNA extraction at 0d, 1d, 2d, 3d and 4d after inoculation. One hundred thrips were used for each DNA sample. There were three replicate samples for each temperature treatment at each time point.

#### Extraction of *B. bassiana* DNA from infected thrips

Groups of 100 thrips were placed in grinding tubes containing 0.25 g of zirconium beads (diameter: 0.2 mm) and 0.25 g of silica beads (diameter: 0.8 mm) and macerated with a Tissue Lyzer^TM^ (Qiagen) tissue grinder for 1 min at 30 HZ. During maceration, grinding tubes were re-adjusted every 15 s to ensure that fungi in each tube were fully ground. Then, 600 μL of nuclear lysate was added to each of tube, and grinding continued, as described above. After maceration, the mixed DNA of *B. bassiana* and *F. occidentalis* was extracted by using Wizard® Genomic DNA Purification Kit (Promega, USA) and then dissolved with Nuclease-Free Water.

#### Standard curve and sample quantification

1 ml of a conidial suspension (1 × 10^8^ conidia per milliliter) prepared with sterile water and 0.05% Tween-80 was centrifuged. The supernatant was discarded, and the DNA, extracted as described above, was used as a standard. Serial dilutions of the standard, ranging from 1 × 10^8^ to 1 × 10^2^ conidia per milliliter) were prepared. A standard curve was generated by running three replicates of each serial dilution through qPCR runs. Based on the volume of DNA in each run, which was equated to the equivalent of the DNA extracted from a single spore, the limit of detection was the amount of DNA from 100 conidia (2 μl/200 μl).

Primers and probes were designed for real-time fluorescence quantitative PCR based on the ITS2 and ribosomal RNA (rRNA) sequences of *B. bassiana* accession no. AF345539 (GenBank), according to techniques described by Bell *et al*.^34^. The upstream primer was: 5′-GCCGGCCCTGAAATGG-3′, and the downstream primer was: 5′-GATTCGAGGTCAACGTTCAGAAG-3′; and the Probe was: 6-FAM-ACAGCTCGCACCGGA-MGB. Real-time fluorescence quantitative PCR runs were conducted on an Applied Biosystems 7500 Real-Time PCR System: pre-denaturation at 95 °C for 2 min, followed at 95 °C for 15 s, and then 40 cycles at 60 °C for 1 min. The operating system was 20 μl: 4.8 μl of Nuclease-Free Water, 10 μl of TaqMan Universal Master Mix II, no UNG, 1.2 μl of the upstream primer, 1.2 μl of the downstream primers, 0.8 μl of the probe, and 2 μl of DNA sample from the conidia.

Quantification of extracted *B. bassiana* DNA from infected thrips was accomplished by comparing threshold cycle numbers against the standard curve. The extraction of *B. bassiana* DNA from infected thrips and following qPCR system were the same as described above. The standard curve was obtained by measuring the amplification curve of seven serial dilution points (10^−8^–10^−2^) of the standard. The resulting standard curve was calculated as:

$$y=-\,3.353x+35.864\,({R}^{2}=0.998),$$

where *y* = cycle threshold and *x* = log(initial DNA concentration).

The amplification efficiency was 100–102%.

### Statistical analyses

To determine temperature preferences of infected and healthy thrips, the proportions of thrips located in each temperature zone were analyzed by ANOVA with fungal treatment as the independent variable. Proportions were arcsine-square root transformed before analysis.

Cumulative adult survival after treatment was calculated daily by subtracting the number of dead adults from the initial number of tested individuals in each treatment and temperature replicate. The number of total viable eggs per female was subjected to analysis of variance (ANOVA), and differences among treatments were compared using Tukey’s test (*P* < 0.05). Differences in sex ratios among groups according to temperature (12 or 24 °C) and fungal-infection were assessed with analysis of variance (ANOVA) followed by Tukey’s test. All data presented here are mean ± standard error (SE). All statistical analyses were performed in SPSS^35^, and significance was set at *P* ≤ 0.05.

A two-way ANOVA was constructed to test for the effects of temperature and sampling time and their interaction on the copy number of fungal genes in thrips. The gene copy numbers were logarithmically transformed (Log_10_[number of copies + 1]) before analysis.

## Results

### Temperature preference of thrips

**Figure 1:**
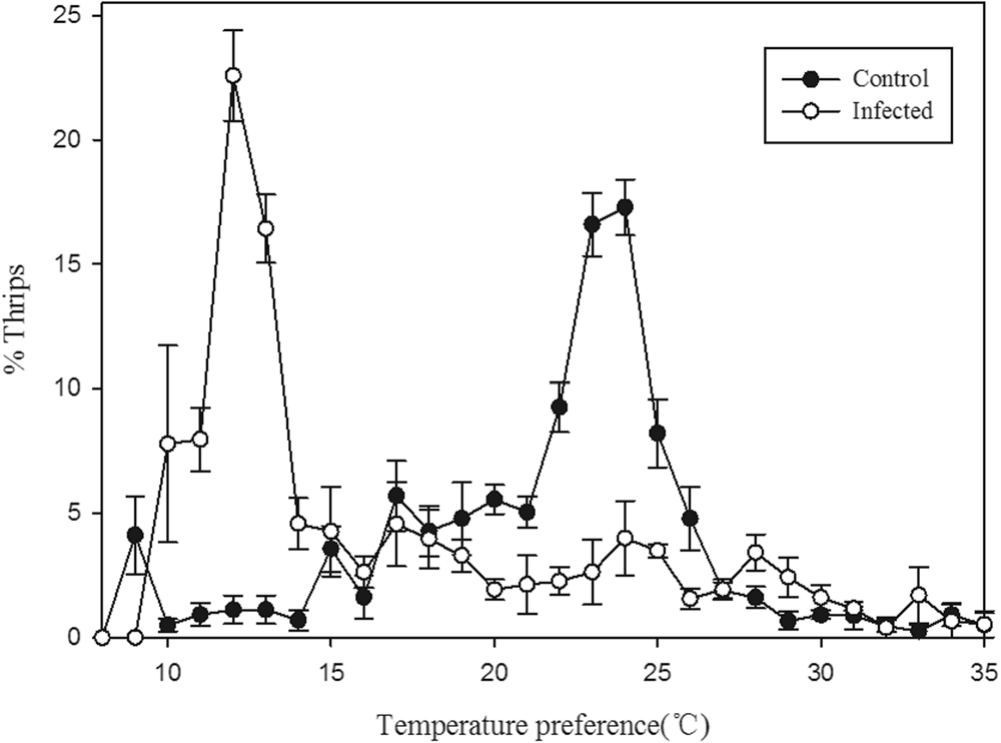
Percentage of thrips selecting temperature points along an aluminum board with a thermal gradient ranging from 8–35 °C. *Frankliniella occidentalis* infected with *Beauveria bassiana* prefer colder temperatures relative to healthy, control thrips. 24 h following exposure to *B. bassiana*, infected thrips preferred 12 °C, but healthy thrips preferred 24 °C. All data represent means ± SE.

*Beauveria bassiana-*infected thrips preferentially moved to colder temperatures along the thermal gradient than did healthy thrips. Infected thrips preferred 12 °C, whereas the preferred temperature for healthy thrips was ~24 °C (Fig. 1; Control: F = 33.66, *P* < 0.0001) 17.28% of thrips settled at 24 °C and 16.59% settled at 23 °C. The most preferred temperature for infected thrips was 12 °C. (F = 15.78, *P* < 0.0001). These results indicate that the differential movement of thrips response to fungal infection are consistent with a chilling behavioral response.

### Effect of chilling on the survival rate and sex ratio of parental thrips

**Figure 2:**
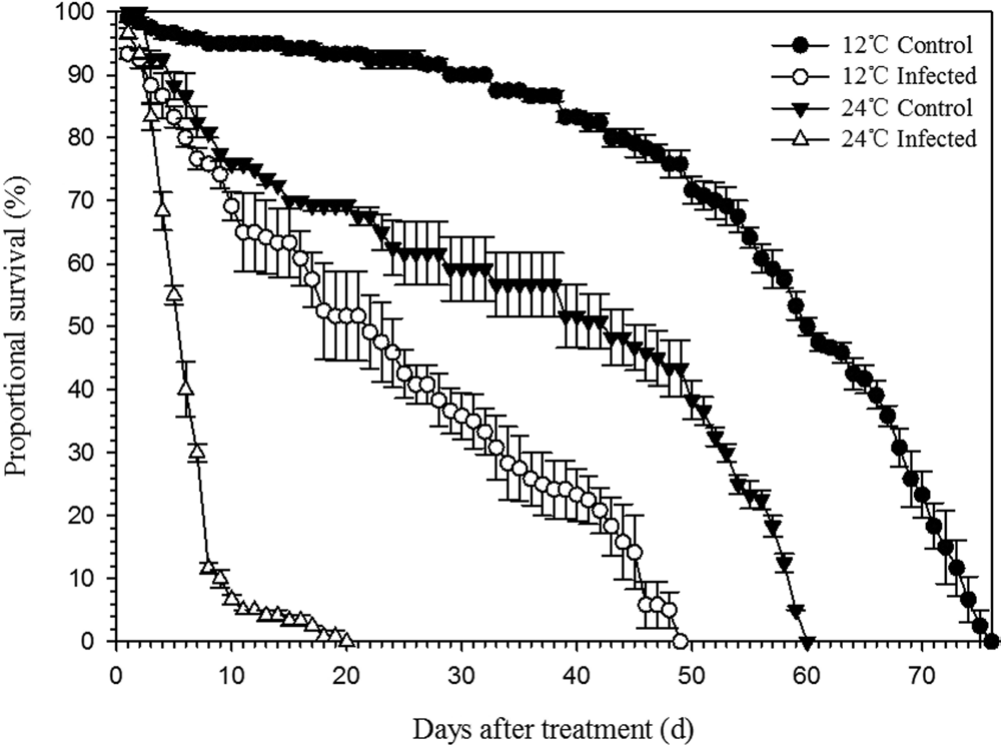
Effect of temperature (12 °C and 24 °C) on survival of infected and uninfected adult *F. occidentalis*. Data represent the proportion of surviving *F. occidentalis* adults that were either infected with *B. bassiana* or not infected upon adult eclosion, and then were held, respectively, at 12 °C and 24 °C. All data represent means ± SE.

Regardless of temperature (12 °C or 24 °C), infection by *B. bassiana* significantly reduced survival rates of *F. occidentalis*. However, *B. bassiana-*infected thrips had a higher survival rate and longer lifespan when held at 12 °C than when held at 24 °C. Although the colder temperature prolonged the life cycle of infected thrips, it did not prevent their ultimate mortality induced by the fungus. The lifespan of healthy thrips also was greater at the lower temperature than at the higher temperature (Fig. 2).

**Figure 3:**
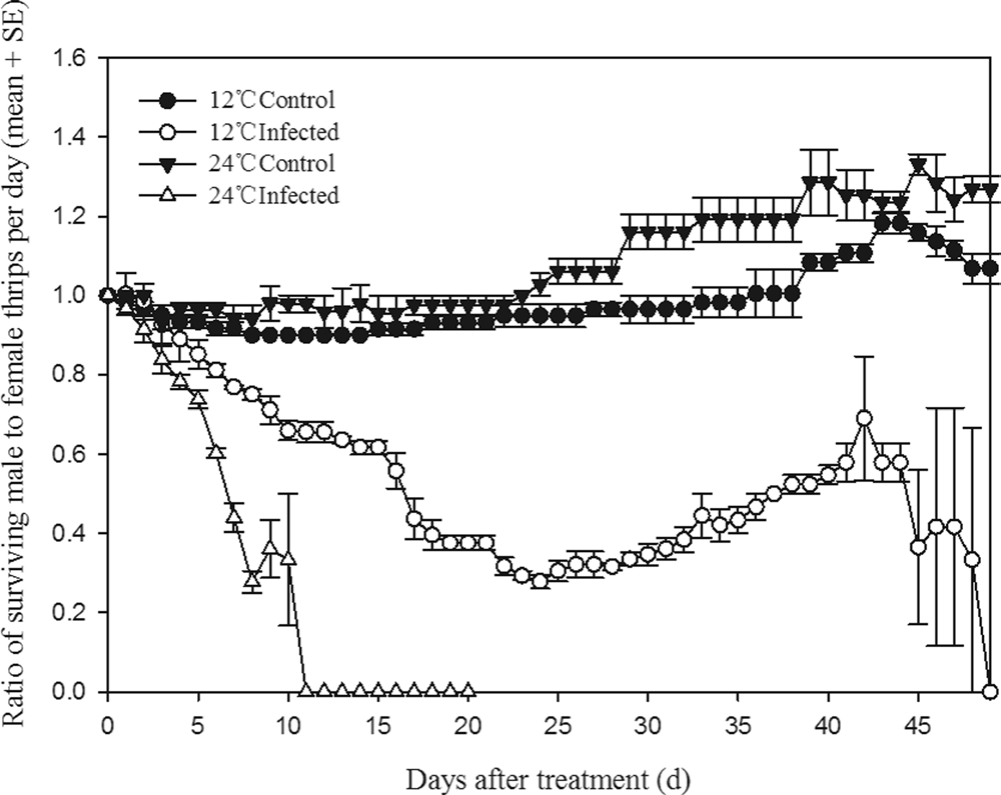
Effect of temperature and *B. bassiana* infection on the sex ratio (♂:♀) of surviving thrips. All data represents means ± SE.

At 24 °C, the sex ratio (♂:♀) of surviving infected thrips decreased over time from day 1 to day 11. All males had died by day 11 (Fig. 3), and the last female survived until day 20 (Figs 2, 3). At 12 °C, the sex ratio (♂:♀) of infected thrips changed over time (day 1 to day 49). The maximum life span for males was as long as for females, with individuals of both sexes surviving 49 days (Figs 2, 3).

Probit analyses showed that temperature significantly affected survival of infected thrips. Survival at 12 °C was longer than that at 24 °C, based on their respective 95% fiducial limits for 50% lethal times not overlapping (12 °C: LT_50,control_ = 61.66 [95% fiducial limits: 60.17–63.19]; 24 °C: LT_50,control_ = 32.94 [30.16–36.18]). Regardless of temperature (12 °C or 24 °C), uninfected thrips lived longer than infected individuals (12 °C: LT_50,control_ = 61.66 (95% fiducial limits: 60.17–63.19), LT_50,infected_ = 17.37_,_ (15.85–18.91); 24 °C: LT_50,control_ = 32.94 (30.16–36.18), LT_50,infected_ = 4.86 (4.56–5.16).

The sex ratio (♂:♀) of control thrips at 24 °C was slightly higher than that at 12 °C, but at both temperatures the sex ratio remained near unity, indicating that survival rates of uninfected males was similar to those of uninfected females. Taken together, these results indicate that infection by *B. bassiana* is more virulent to male *F. occidentalis* than it is to female *F. occidentalis*.

### Effect of temperature and *B. bassiana* infection on fecundity and sex ratio of next generation

**Figure 4:**
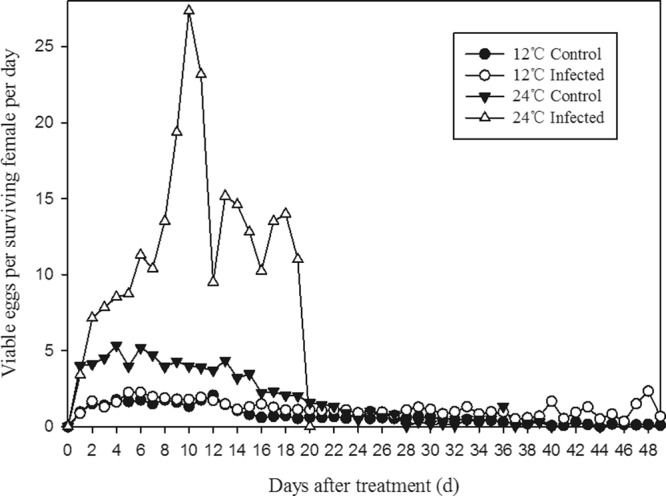
Mean daily number of viable eggs per surviving female of *B. bassiana-*infected and uninfected (control) *F. occidentalis* maintained at 12 °C and 24 °C. Newly emerged adults of *F. occidentalis* (20 pairs) were treated with *B. bassiana* or 0.05% Tween-80 and then maintained at either 12 °C or 24 °C.

**Figure 5:**
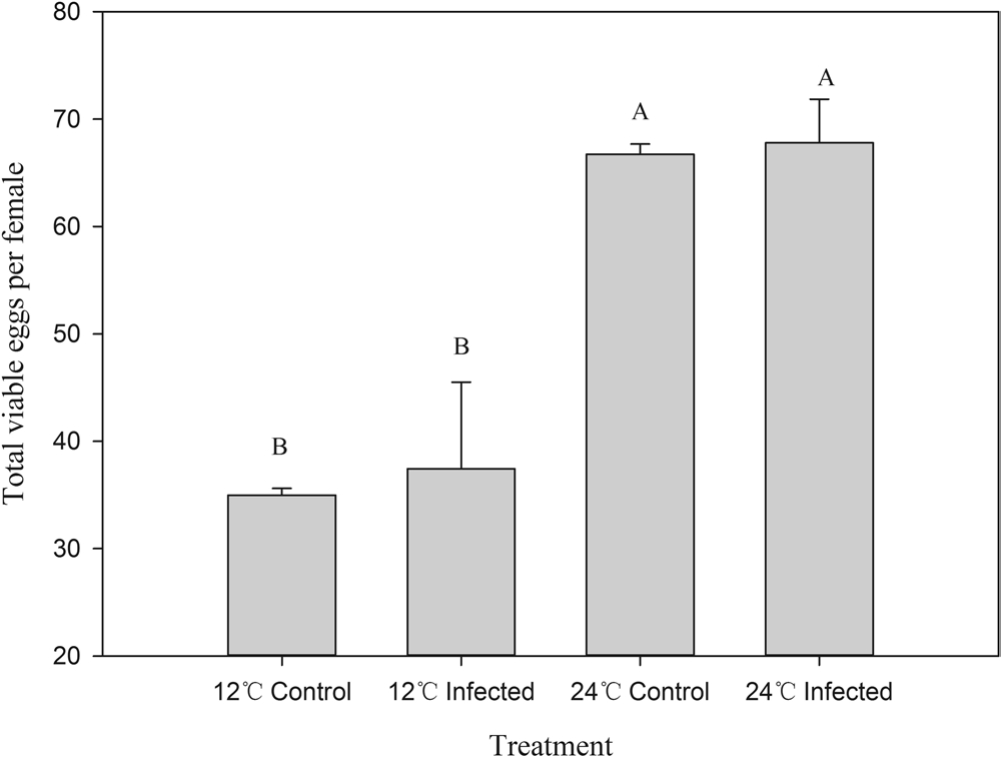
Mean total viable eggs laid by *B. bassiana-*infected and uninfected (control) female *F. occidentalis* maintained at either 12 °C or 24 °C. Different letters indicate significant difference at *P* < 0.05, Tukey’s test. All data represent means ± SE.

At 24 °C, fungal infection led to significant changes in the reproductive success of *F. occidentalis*. Infected females tended to lay more eggs at early ages, with multiple peaks of oviposition compared with uninfected females. However, the survivorship of infected females was lower than that of uninfected females (Fig. 4). Because of the differences in survival, there was no significant difference in total numbers of eggs laid between infected and uninfected females (Fig. 5).

At 12 °C, there was no difference in the mean number of viable eggs laid by infected females and uninfected females early in the adult stage. Later in the adult stage, fecundity of infected females was higher than that of uninfected females (Fig. 4). However, total lifetime fecundity did not differ between uninfected and infected females.

Infected thrips maintained at 24 °C had significantly higher early-age fecundity at the expense of lower late-age egg production than those infected and maintained at 12 °C, which had higher late-age fecundity (Fig. 4). At the colder temperature (12 °C), total egg production of the infected and healthy thrips populations was lower than for their respective populations maintained at the warmer temperature (Fig. 5; 12 °C Infected = 748 ± 161, 24 °C Infected = 1356 ± 81, 12 °C Uninfected = 699 ± 12, 24 °C Uninfected = 1334 ± 19; F_3,11_ = 4.70, *P* = 0.0355), even though chilling extended survival times (Fig. 2).

At 24 °C, infected thrips had greater early-age fecundity (Fig. 4), but lower survival (Fig. 2) and lifetime egg production (Fig. 5) compared with uninfected thrips.

Infection of *F. occidentalis* parents with *B. bassiana* led to a greater proportion of male progeny compared with the progeny of healthy parents (Table 1) (12 °C: *F* = 208.14, *P* < 0.001; 24 °C: *F* = 257.44, *P* < 0.001). Temperature also had a significant effect on the sex ratio of progeny (Control: *F* = 8.3, *P* = 0.045; Infected: *F* = 27.08, *P* < 0.001). At the colder temperature of 12 °C, there was a significantly greater percentage of female progeny of infected parents (32.0%) than there was at 24 °C (28.4%). However, the percentage of females among the progeny of healthy, control parents at 12 °C was lower (44.4%) than among the progeny of healthy, control parents at 24 °C (49.2%). The survival rate of the progeny was also dependent on temperature. Survival of progeny to adulthood was higher at 12 °C than at 24 °C for progeny of either infected parents or healthy, control parents.

**Table 1:** The sex ratio and survival to adulthood of progeny of parental *F. occidentalis* that had been treated with *B. bassiana* (Infected) and progeny of parental *F. occidentalis* that were not treated with *B. bassiana* (Control), and maintained at 12 °C or 24 °C.

Treatment of the parental *F. occidentalis*	survival rate till adult (mean proportion ± SE)	Sex ratio (♂:♀)
	(the progeny of parental *F. occidentalis*)	
12 °C Control	0.98 ± 0.01	1.26 ± 0.04
12 °C Infected	0.87 ± 0.08	2.09 ± 0.04
24 °C Control	0.88 ± 0.05	1.04 ± 0.06
24 °C Infected	0.80 ± 0.04	2.50 ± 0.07

### *Beauveria bassiana* content in infected thrips

Temperature, time, and the interaction of temperature and time significantly affected the gene copy number of *B. bassiana* recovered from infected thrips (Temperature: *F*_1,29_ = 254.58, *P* < 0.0001; Time: *F*_4,29_ = 253.79, *P* < 0.0001; Interaction: *F*_4,29_ = 47.26, *P* < 0.0001). The interaction resulted from an initial decline in gene copy number from day 0 to day 1, and the subsequent increase in gene copy numbers over the following days. The rate of increase was greater at 24 °C than at 12 °C.

**Figure 6:**
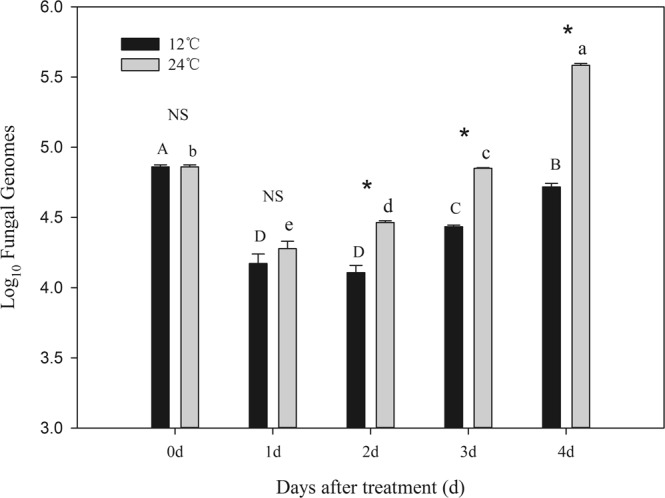
Fungal counts, as measured by gene copy numbers, recovered from live *F. occidentalis* that had been treated with *B. bassiana* (100 thrips per replicate). Different upper case letters indicate significant differences in *B. bassiana* levels among days after treatment for *F. occidentalis* maintained at 12 °C, and different lower case letters indicate significant differences in *B. bassiana* levels among days after treatment for *F. occidentalis* maintained at 24 °C (*P* < 0.05, Tukey test for all means comparisons). Asterisks represent significant differences in *B. bassiana* levels between temperature treatments for each day after treatment. NS indicates no significant difference between temperature treatments within days (*P* > 0.05). All data represent means ± SE and are on a logarithmic scale.

At each temperature, time after inoculation had significant effect on the gene copy number of the fungi *in vivo*. Although copy numbers declined from the day of treatment to the day after treatment, at both temperatures, fungal counts increased subsequently. However, fungal growth rates after day1 were much greater at 24 °C than that at 12 °C (Fig. 6). At 12 °C, gene copy number significantly increased after day 2 through day 4 (*F* = 66.13, *P* < 0.0001). At 24 °C, gene copy number significantly increased after day 1 through day 4 (24 °C: *F* = 379.34, *P* < 0.001). There was no significant difference between temperatures in gene copy numbers immediately following treatment (0 d, Fig. 6). However, by the second day after treatment, gene copy number of the fungi *in vivo* at 12 °C was significantly lower than that at 24 °C (1d: *t*_4_ = −1.24 *P* = 0.2832; 2d: *t*_4_ = −6.69 *P* = 0.0026; 3d: *t*_4_ = −34.76 *P* < 0.0001; 4d: *t*_4_ = −30.72, *P* < 0.0001).

## Discussion

Temperature plays a significant role in the interactions between hosts and their parasites or pathogens^28^. Even small herbivorous insects can experience a wide range of temperatures and microenvironments within very short distances of a few cm of a plant canopy^36^ indicating that thermoregulation could be a realistic defensive mechanism for thrips to employ. Even small changes in temperature may differentially benefit hosts at the expense of their parasites or pathogens^28^. The findings of our study are consistent with this idea in that colder temperatures help thrips survive and reproduce following fungal infection.

We found that *F. occidentalis* infected with *B. bassiana* actively sought out cooler temperatures whereas healthy thrips preferred significantly warmer temperatures. These differences indicate that infection by *B. bassiana* leads *F. occidentalis* to alter its behavior. Behavioral thermoregulation has been reported in a wide diversity of insects when infected with parasitic or pathogenic organisms^4,13,18,21,28^. However, these thermoregulatory responses may either be to the benefit of the parasitic or pathogenic organism, or to the benefit of the host organism^37^. The question of which organism, if either, may benefit from changes in thermoregulatory behaviors of the host is complex and should be resolved by examining how the behaviors affect the life histories of the species involved.

Thermoregulatory responses by a host species may also be specific to the particular parasitic or pathogenic species in the relationship. The same host species may thermoregulate in different manners when infected by different species of parasites, such as *Planorbarius corneus* does when parasitized by different species of trematode worms^38^. In some cases, a host species may thermoregulate in response to some parasite species but not in response to attack by other pathogenic or parasitic species^13^. Behavioral responses may also vary among host species when attacked by the same parasite or pathogen. For example, infection by *B. bassiana* induces behavioral fevering in *Melanoplus sanguinipes*, which reduces fungal development in the host^39^. However, in our study, *F. occidentalis* responded to *B. bassiana* infection by behavioral chilling.

The colder temperature of 12 °C preferred by infected thrips greatly improved their survival compared with the warmer temperature of 24 °C preferred by healthy thrips. This chilling response may be due to suppression of the growth of *B. bassiana in vivo* in colder temperatures. The initial decline in gene copy numbers from day 0 to day 1 may be the result of the loss of applied *B. bassiana* from the body surface of *F. occidentalis*. The greater amounts of *B. bassiana* gene copy numbers detected at later times likely represent fungi that had established infections and begun to reproduce. From the second day after application onward, the gene copy number for *B. bassiana* was significantly higher at 24 °C than at 12 °C, and the difference increased over time, suggesting much greater fungal population growth at the higher temperature. By 4 days after treatment, there was approximately a 10-fold difference in gene copy numbers between thrips held at the two different temperatures. Cold temperatures are known to inhibit the growth and virulence of entomopathogenic fungi attacking *Myzus persicae*^40^, *Monochamus alternatus*^41^, *Megalurothrips sjostedti*^42^ and *Chilo partellus*^43^.

In addition to reducing fungal population growth, lower temperatures also may reduce the ability of entomopathogenic fungi to utilize host resources^44^. Lower temperatures can increase stress tolerances of insects, with lower temperatures increasing expression of immunity-related genes^37,45,46^. For example, at 29 °C, the fruit fly *Drosophila melanogaster* only displays up-regulation of the *Metchnikowin* (*Mtk*) gene. However at lower temperatures, additional immune pathways are upregulated. At 25 °C, *Mtk* and *Peptidoglycan recognition protein-LC* (*Pgrp-LC*) genes are upregulated, and at 17 °C, *Cactus* (*Cact*) is upregulated, as well as *Mtk* and *Pgrp-LC*^37^. *F. occidentalis* also appears to have low-temperature-induced immune expression pathways (unpublished data), and we are examining the effect of chilling temperature on the immune gene expression of the infected thrips.

Because insects are ectothermic organisms, longevity is often inversely related to temperature, within physiological limits, for many insects^47^, including those cold-seeking, infected ones, such as *Drosophila*^18^. For the infected thrips in our study, exposure to the preferred chilling temperature increased survivorship, especially the survivorship of males. Because of the arrhenotokous reproductive in *F. occidentalis*, longer-term survivorship of males may increase the ability of females to produce female progeny. We did not observe differences in total reproductive success between infected and healthy thrips at either of the tested temperatures, although reproduction was greater at 24 °C than at 12 °C. Although infected females laid fewer eggs at 12 °C than at 24 °C, survivorship of progeny to adulthood was greater at the lower temperature.

We did observe large effects of fungal infection and temperature on the sex ratio of progeny. Progeny from infected parents were more male-biased than were progeny from healthy parents. Zhang *et al*.^27^ have also shown that the offspring of *F. occidentalis* infected by *B. bassiana* are more male-biased than are progeny from healthy parents. It is possible that females respond to infection by *B. bassiana* by laying a greater proportion of unfertilized eggs, and thus produced more male offspring, or it may result from fungal effects on male parental thrips. The production of viable sperm may have been compromised in treated males, and this effect may have been amplified at the higher temperature. The progeny of infected parents at 12 °C was much less male-biased than progeny of infected parents at 24 °C, which was the opposite pattern than for progeny from healthy parents. The relationship between progeny sex ratio and temperature has also been reported by Kumm & Moritz (2009)^26^. The increased proportion of females at the lower temperature among progeny from infected parents suggests that chilling behavior is beneficial for *B. bassiana-*infected *F. occidentalis*.

To date, many studies evaluating entomopathogenic fungal growth in thermoregulating insects have only considered growth *in vitro*, on artificial media^6,48,49^. However, these methods may adequately represent the conditions to which fungi are subjected. Such *in vitro* conditions may lack the same immune pressures and nutritional environment that would be present in a host insect. In turn, microscopy techniques to quantify fungal populations may not accurately account for fungal growth within the bodies of host insects^4,18^. We were able to overcome these limitations by assessing thermal effects on *B. bassiana* within a host species and utilizing quantitative PCR (qt-PCR) protocols to quantify the growth of fungi in a thermoregulating host insect.

In conclusion, we propose that *F. occidentalis* respond to infection by *B. bassiana* by engaging in chilling behavior. Chilling behavior of thrips suppresses the growth of *B. bassiana* greatly improves the survival of infected individuals. Lower temperatures also appear to be beneficial for the reproductive success of *B. bassiana* infected *F. occidentalis*. While behavioral fevering may be beneficial for some insects to counteract *B. bassiana* infection, there may be important ecological reasons for *F. occidentalis* to respond with behavioral chilling. Small insects such as *F. occidentalis* are extremely susceptible to desiccation from high temperatures^49–51^. Lethal temperatures for *F. occidentalis* are at or below lethal temperatures for *B. bassiana*^41,42,50^.

Therefore, chilling behaviors would be more adaptive for *F. occidentalis* than behavioral fever would be. The behavioral thermoregulation of host insects play an important role in an insects response to challenges from pathogens and parasites, and may significantly influence the outcome of management practices employing these types of beneficial organisms.
